# Applying genomics in regulatory toxicology: a report of the ECETOC workshop on omics threshold on non-adversity

**DOI:** 10.1007/s00204-023-03522-3

**Published:** 2023-06-09

**Authors:** Timothy W. Gant, Scott S. Auerbach, Martin Von Bergen, Mounir Bouhifd, Philip A. Botham, Florian Caiment, Richard A. Currie, Joshua Harrill, Kamin Johnson, Dongying Li, David Rouquie, Ben van Ravenzwaay, Frank Sistare, Tewes Tralau, Mark R. Viant, Jan Willem van de Laan, Carole Yauk

**Affiliations:** 1 United Kingdom Health Security Agency, Harwell Science Campus, Didcot, Oxfordshire United Kingdom; 2 Imperial College London School of Public Health, London, United Kingdom; 3 Division of Translational Toxicology, National Institute of Environmental Health Sciences, RTP, Durham, NC USA; 4 Department for Molecular Systems Biology, Helmholtz Centre for Environmental Research, Leipzig, Germany; 5 European Chemicals Agency, 00121 Helsinki, Finland; 6 Syngenta, Jealott’s Hill, Bracknell, Berkshire UK; 7 Department of Toxicogenomics, Maastricht University, Maastricht, The Netherlands; 8 Cellular and Molecular Toxicologist, Center for Computational Toxicology and Exposure (CCTE), U.S. Environmental Protection Agency, Durham, NC USA; 9 Predictive Safety Center, Corteva Agriscience, Indianapolis, IN USA; 10 National Center for Toxicological Research, U.S. Food and Drug Administration (FDA), 3900 NCTR Road, Jefferson, AR 72079 USA; 11 Bayer SAS, Bayer Crop Science, 355 Rue Dostoïevski, CS 90153, 06906 Valbonne Sophia-Antipolis, France; 12 Environmental Sciences Consulting, 67122 Altrip, Germany; 13 University of North Carolina, Chapel Hill, NC USA; 14 Department of Pesticides Safety, German Federal Institute for Risk Assessment (BfR), Berlin, Germany; 15 School of Biosciences, University of Birmingham, Birmingham, UK; 16 Medicines Evaluation Board, Utrecht, The Netherlands; 17 Department of Biology, University of Ottawa, Ottawa, Canada

**Keywords:** PODS, Transcriptomics, Workshop, ECETOC

## Abstract

In a joint effort involving scientists from academia, industry and regulatory agencies, ECETOC’s activities in Omics have led to conceptual proposals for: (1) A framework that assures data quality for reporting and inclusion of Omics data in regulatory assessments; and (2) an approach to robustly quantify these data, prior to interpretation for regulatory use. In continuation of these activities this workshop explored and identified areas of need to facilitate robust interpretation of such data in the context of deriving points of departure (POD) for risk assessment and determining an adverse change from normal variation. ECETOC was amongst the first to systematically explore the application of Omics methods, now incorporated into the group of methods known as New Approach Methodologies (NAMs), to regulatory toxicology. This support has been in the form of both projects (primarily with CEFIC/LRI) and workshops. Outputs have led to projects included in the workplan of the Extended Advisory Group on Molecular Screening and Toxicogenomics (EAGMST) group of the Organisation for Economic Co-operation and Development (OECD) and to the drafting of OECD Guidance Documents for Omics data reporting, with potentially more to follow on data transformation and interpretation. The current workshop was the last in a series of technical methods development workshops, with a sub-focus on the derivation of a POD from Omics data. Workshop presentations demonstrated that Omics data developed within robust frameworks for both scientific data generation and analysis can be used to derive a POD. The issue of noise in the data was discussed as an important consideration for identifying robust Omics changes and deriving a POD. Such variability or “noise” can comprise technical or biological variation within a dataset and should clearly be distinguished from homeostatic responses. Adverse outcome pathways (AOPs) were considered a useful framework on which to assemble Omics methods, and a number of case examples were presented in illustration of this point. What is apparent is that high dimension data will always be subject to varying processing pipelines and hence interpretation, depending on the context they are used in. Yet, they can provide valuable input for regulatory toxicology, with the pre-condition being robust methods for the collection and processing of data together with a comprehensive description how the data were interpreted, and conclusions reached.

## Introduction

Transcriptomics data have long been suggested to be of added value in toxicology to complement in vivo and in vitro evaluations in the context of hazard characterization and risk assessment of chemicals. The main advantage of such evaluations lies in the large amount of biological information generated, which provides a global overview of the altered gene expression levels in a given biological sample following chemical exposure. Thereafter, the challenge is to translate the generated biological information into biological knowledge that can be used for regulatory decision making. This paper summarizes the outcomes of an ECETOC workshop addressing the obstacles impeding the regulatory use of transcriptomic data.

At the start of this millennium, the first reports using microarrays were published, evolving from cDNA clones on nylon (Bertucci et al. 1999), to cDNA probes on glass, and finally oligonucleotide microarrays technologies. The latter format harnessed the knowledge of the whole genome published first in 2003 2003 (Cheung et al. 1999; Lander et al. 2001; Schena et al. 1998).

In the early development of these technologies, there were many anticipated applications to advance regulatory toxicology capability and capacity (Corton et al. 1999; Nuwaysir et al. 1999). Three major advancements that were envisioned for Omics in regulatory toxicology included: (1) providing powerful replacements for traditional toxicological testing by delivering more information on mechanisms and predicting outcomes; (2) improving the delineation and stratification of pathological outcomes (refinement) (Golub et al. 1999); and (3) reducing the duration of animal testing by using Omic changes indicative of the adverse outcome rather than waiting for the cellular and organ changes to manifest. For example, metabolomics and multiomics-based testing theoretically could reach the same conclusion as a rat 2-year carcinogenicity study using only a short-term exposure (Ellinger-Ziegelbauer et al. 2008).

Today other Omics technologies, particularly RNA sequencing, have largely replaced microarrays except for niche applications such as comprehensive functional arrays. Nonetheless, a repository of microarray data exists that will be of value for years to come. Metabolomics has undergone a similar revolution, moving from NMR-based technology to a data generation pipeline largely based on mass spectrometry techniques. For both transcriptomics and metabolomics, there have been parallel advances in bioinformatics analysis necessary to process and interpret the large amounts of information generated. Without this bioinformatic processing power, analysis of the data generated from these Omics technologies would be possible only at the most superficial level (Kanehisa and Bork 2003; Salter and Nilsson 2003). From these beginnings, other Omic methods were developed to examine cellular biomolecules in high-throughput format including proteomics, lipidomics, and variety of methods for DNA and RNA sub-types (e.g., modifications for epigenetics and small non-coding RNA) (Joyce and Palsson 2006). The latter methods are not discussed further in this report because, though important in research, they are the least developed for regulatory purposes. With further development, these other ‘omic molecular methods may find important future application in regulatory toxicology.

Omics are one member of a group of methods called New Approach Methodologies (NAM), which are in vivo or in vitro methods that essentially enhance the pace of work (provide data more quickly), contribute mechanistic understanding (European Chemicals Agency 2016; USEPA 2018). Importantly NAMs can be used to refine, reduce and replace animal use. Innovations in technology and establishment of basic principles in study design have led to cost reductions and increased efficiencies in data processing. This provides the opportunity to apply these methods more widely in chemical risk assessment particularly to generate initial stratification and decision data within a tiered risk assessment strategy, where the testing required within each tier is informed by data from the previous tiers with in vivo use only occurring within the higher tiers. Building consensus to identify the optimal and acceptable methods for Omics applications in regulatory toxicology has been a focus of ECETOC collaborative work over the last 7–8 years. The aim has been to gain a wider acceptance of ‘omics methods and inclusion into the portfolio of methods that can be applied to chemical regulatory toxicology within a tiered testing framework that places a much greater emphasis on decreasing animal use (Ball et al. 2022). The workshop described here examined what has been achieved in the Omics technical space and looked forward to the use of Omics data to estimate toxicological points of departure (POD) that can be used in chemical assessments.

## Workshop background

**Fig. 1:**
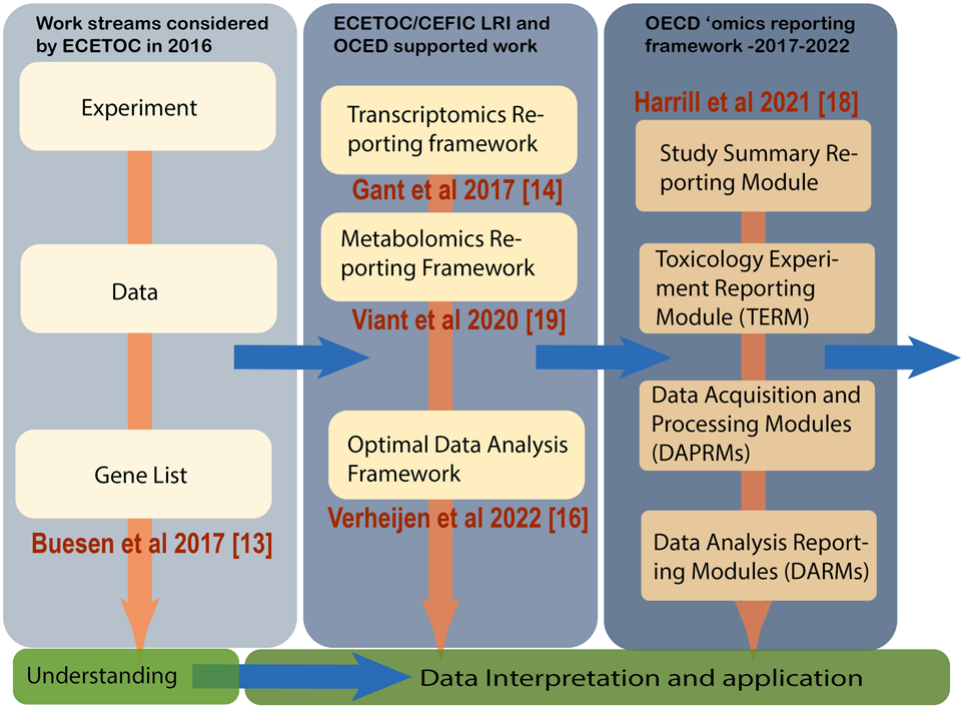
Progress from the initial concept to the Omics reporting framework and optimal data analysis framework. Shown in yellow is the understanding and interpretation that has been outside this area of activity but recognised as an area requiring focus going forward

The European Centre for Ecotoxicology and Toxicology of Chemicals (ECETOC) has a long involvement in facilitating the development of Omics methods both alone and with the CEFIC/LRI program. The work to start examining the process of data transformation in Omics that led to the meeting reported here began in 2015, with a consideration of how to transform the data prior to interpretation from a Cefic Long-range Research Initiative (CEFIC/LRI) project called Combined Low-Dose Exposures to Anti-Androgenic Substances (EMSG56). This project was a pre–postnatal in vivo study in rats that generated in total 120 Agilent microarrays and is described in full in the Supplementary section. During the bioinformatic processing of these data it was realized that there were no internationally accepted methods for the data transformation from the raw data to the differential gene expression list (Buesen et al. 2017; Sauer et al. 2017). This provided the incentive for a series of workshops in 2015 and 2016 that led to the development of the concept of an Optimal Data Analysis Framework, which was published in 2017 as the Reference Baseline analysis (Gant et al. 2017). A call for further development of this Reference Baseline analysis was made in the C4 CEFIC/LRI call (https://cefic-lri.org/projects/c4-transcriptomics-bioinformatics-best-practices-in-toxicogenomics-for-regulatory-application-2/). This was taken forward by the University of Maastricht and renamed the Omics Data Analysis Framework (ODAF) (Verheijen et al. 2020). The outcome of this work and associated R code was published in 2022 (Verheijen et al. 2022). In 2016, ECETOC sponsored another workshop entitled ‘Applying Omics technologies in Chemical Risk Assessment’ (Sauer et al. 2017). This workshop led to the proposal to develop two reporting frameworks for Omics data, capturing transcriptomics and metabolomics (Fig. 1). The two reporting frameworks were presented to the Extended Advisory Group on Molecular Screening and Toxicogenomics (EAGMST) group of the OECD by Timothy W. Gant and Miriam Jacobs (UK National Co-ordinator for Human Health to the OECD) in June 2016 using a Standard Project Submission Form. After further refinement, including input from the US Environmental Protection Agency (US EPA) and Health Canada, the project was accepted onto the workplan in December 2017 and led by the USA, Canada and the UK. During the EAGMST supported project, the TRF and MRF were harmonized into a single OECD Omics Reporting Framework (OORF) document that contains; (1) toxicology Experiments reporting module (first data entry module) that summarizes either the animal or in vitro toxicology experimental work that was done, (2) Data Acquisition and Processing Report Modules (DAPRM) specific for particular transcriptomics and metabolomics technologies; and (3) Data Analysis Reporting Modules (DARMs) harmonized where possible to facilitate reporting of any type of Omics data. From this work, a paper has been published describing the transcriptomics and metabolomic reporting modules, together forming the OORF (Fig. 1). (Harrill et al. 2021b; Viant et al. 2019). The OORF was accepted by the EAGMST group.

This workshop built on the former initiatives and was designed specifically to explore the final hurdles that need to be overcome to gain further regulation acceptance of the application of the Omics methods in chemicals regulation, with a specific focus on the utility of points of departure (PODs) in Omics data.

The workshop was spread over two days with the first day being given to prepared presentations in two sections: (1) The regulatory perspective; and (2) presentation of the case studies and frameworks for the use and application of Omics data in chemicals regulation. The second day was spent in breakout groups discussing a series of questions presented in the Table 1.

**Table 1:** Questions considered by the breakout groups on Day 2

1	What is a relevant point of departure for different Omics approaches and how is this accurately determined?
2	Given that classical toxicological determination of adversity relies on clinical and pathological endpoints—how do we determine biological significance/adversity of an Omics response or ‘molecular mechanistic response’ (again, against a background of normal biological variation)?
3	What kind of experiment/data set would be necessary to identify non-adverse biological variation of controls? Would it be necessary to determine this for each individual experimental set-up i.e., equivalent to defining normal ‘baseline’ ranges for analytes on a blood clinical chemistry panel?
4	Would mapping basic cellular responses in terms of their Omics-profile (or ‘molecular mechanistic data’) be helpful to better determine a true response from normal biological variation? If yes, how one would ideally proceed with this?

## Workshop presentations

### The regulatory perspective

The regulatory section seeded the scientific perspective that was to follow and was provided by Tewes Tralau (German Federal Institute for Risk Assessment), George Kass (European Food Safety Authority), and Mounir Bouhifd (The European Chemicals Agency). These presentations specially addressed the question of regulatory requirements.

The goal of chemical regulation is protection of human and animal health in addition to environmental protection. To achieve these goals regulatory toxicology relies on hazard and risk assessments codified in science-based legal frameworks. These frameworks support a scientifically defensible justification for the best possible level of health protection and do so in a transparent and legally sound way. Specifically, the conclusions from test methods must provide legal certainty, be based on internationally accepted methods e.g., OECD test guidelines and have earned the trust of regulators through prior use. For these reasons, regulatory methods have tended to focus on defined endpoints for adversity that often have parallels in human pathophysiology which are observed clinically (e.g., histopathological change, tumour formation) and hence have plausible translational relevance to humans and in most cases have a high certitude of adversity. To manifest such endpoints a model system requires the capacity to largely replicate the entirety of biological, physiological and anatomic complexity of the human organism, hence the extensive use of whole organism mammalian model systems such as mice and rats in regulatory testing.

Animal-based model systems will respond in a dose-dependent manner and with a specific apical endpoint (e.g., cell or organ-level pathological alteration) allowing both the identification of the nature of the hazard and a POD, allowing for no-effect levels to be established and the development of classification by hazard and regulation either by hazard or risk where the human exposure is known or can be modelled. The detailed molecular pathways that connect the exposure to the outcome is relevant only in the case that there is a need to understand whether the toxicity observed, and occasionally the dose response, is relevant to humans. It is usually not necessary to understand all the molecular details of the pathway to understand human relevance but only those key elements that are important in connecting exposure to the apical outcome. It is from this recognition that the concept of the Mode of Action (MoA) for a specific chemical and Adverse Outcome Pathway (AOP) as a generic pathway arose as a means of mapping the Molecular Initiating Event (MIE), Key Biological Events (KE) and the causal connections between these events referred to as the Key Event Relationships (KERs).

A key challenge for the application of Omics methods in regulation is that Omics methods are highly dimensional. Highly dimensional means these methods generate data for hundreds or thousands of molecular endpoints; some of the molecular changes observed may be related to the toxicity while other changes may be associated with, or simply result from, the pathophysiology. Therefore, the required analyses and interpretation is more complex than for conventional toxicological tests. An often-applied method to understand these data have been the process of phenotypic anchoring (Moggs et al. 2004; Paules 2003), which matches Omics changes to pathological end points. These approaches can be useful for interpretation, but the latter is usually sufficient on its own for the purpose of regulatory assessment, leaving ‘omics as optional. Omics studies may be required though to build endpoint understanding, with the aim to be able to predict rather than observe the apical endpoint. This is one of the seminal issues for the application of Omics methods in regulation. Unless there is clear agreement on their use or remit, theoretically all of these data might need to be explained for the purposes of regulation and relevance. Scientifically as well as practically, this would be challenging. An understanding is therefore clearly required on what Omics data are relevant for assessing the hazard and this is only likely possible within a adverse outcome pathway-based approach.

Prior to this potential use of Omics, it must be demonstrated that the methods are suitable for regulation and ensure that data generated are consistent, reliable, relevant and provide confidence in the conclusions drawn from the data. Consistency was an early technological issue associated with ‘wet’ procedures but this has somewhat diminished as an issue with increasing technical development. This can be seen in the Cefic LRI EMSG56 data set mentioned above. Specifically, in Gant et al. (2017) Fig. 2, a clear batch effect can be seen in the second of the three data sets. Since the generation of these microarray data, technological improvements, not least of which is the wider use of sequencing technologies, have improved the ‘wet’ procedure variance. Some of the variance associated with the ‘wet’ procedures can be addressed by mathematical transformations that take the raw data to the list of findings for interpretation (e.g., the list of differentially expressed genes). The strength, but also the weakness, of large data sets is that there are numerous mathematical transformations that can be applied, leading to different practitioners using different approaches. While this may be acceptable in research, it is problematic in a regulatory context where consistency and acceptance are key factors. The same arguments apply to reliability, which has improved due to technical advancements but still requires consistent approaches to achieve.

**Fig. 2:**
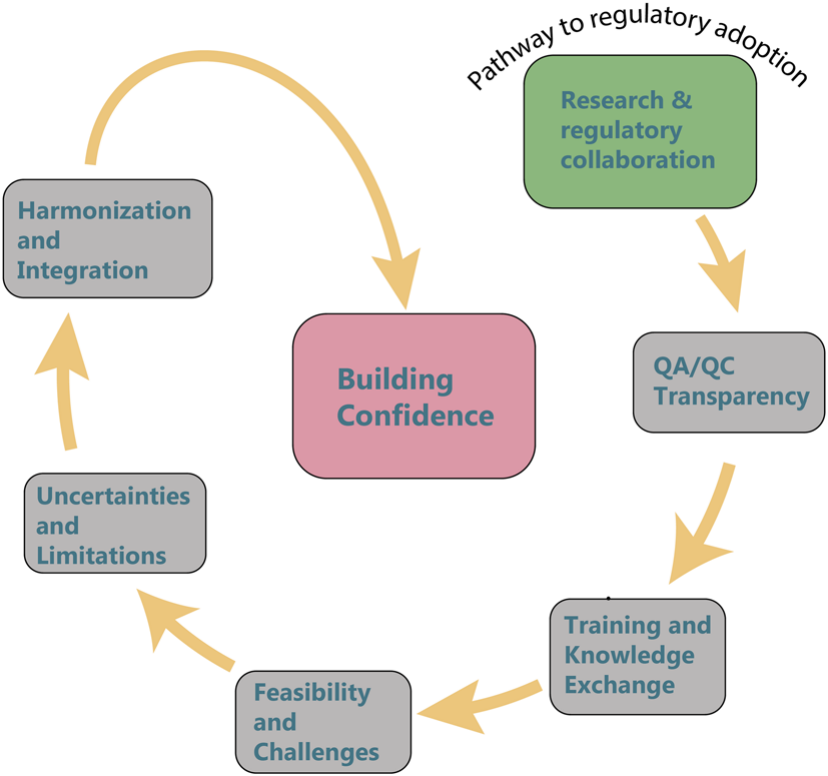
Pathways to regulatory adoption derived from a diagram by Carole Yauk

Omics methods tend now to be comprehensive in their molecular measurements so should by default capture those data which are relevant to the apical outcome. The issue is identifying the relevant data within the landscape. But confidence may be the most significant challenge. When there is the opportunity to apply different transformation methods or interpretations to a large data set, there is reduced confidence in the derived conclusion. This is not so much an issue when there is a binary apical outcome, but Omics methods do not produce binary outcomes. Different practitioners can process the wealth of data in different ways. For regulatory acceptance, therefore, there is a need to provide standardized methods for generating and using Omics data from its generation, reporting, transformation and finally interpretation. This is challenging for single apical endpoint studies; in the landscape of Omics it is even more challenging. This was summarized well in a figure presented by Carole Yauk (Fig. 2).

While the use of Omics in the regulatory assessment of hazard is challenging, particularly within the context of classification, there are good opportunities to use Omics methods in regulatory testing: (1) to better understand the pathway between exposure and apical endpoint and use this information to establish relevance of the model system for human exposure; (2) to identify hazard; (3) to group substances or provide a basis for read-across; and (4) in the future possibly establish protective points of departure below which no toxicological effects are expected.

### Case studies

This session specifically focused on the challenges to be overcome to derive a path forward to facilitate the use of Omics POD data in regulatory submissions and the transformation of data from raw, to that required for interpretation. Presented first in this paper is the data transformation and second the work to derive PODs.

#### The omic data analysis framework for regulatory application (R-ODAF)

**Fig. 3:**
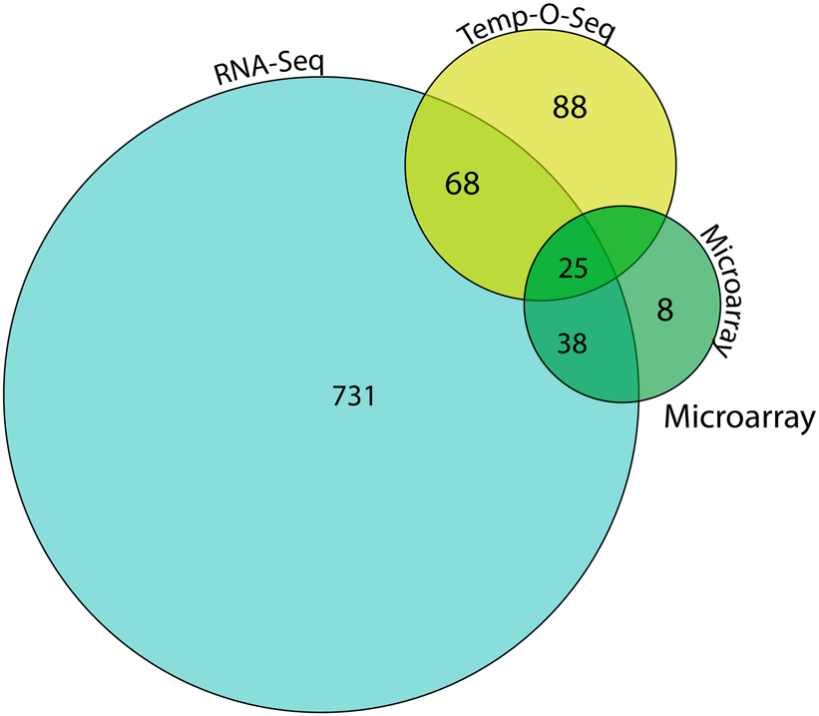
Three Omics methods applied to the same samples with data transformation by the R-ODAF method—redrawn by copyright transfer from (Li et al. 2021)

This work was presented by Florian Caiment (Uni of Maastricht), Dongying Li (US FDA) and Martin von Bergen (Helmholtz University). Caiment presented the R-ODAF that was supported under the CEFIC/LRI project C4 described in the introduction. The method prescribes a series of steps to transform transcriptomic data generated by microarrays, RNA-Sequencing, and TempO-Seq (Templated Oligo-Sequencing) technologies, respectively, and recommends criteria for identifying Differentially Expressed Genes (DEGs). The R-ODAF method has been published (Verheijen et al. 2022) and the R code is available in GitHub (https://github.com/R-ODAF/Main). The point was made that this method addresses a need for a reference point and is not meant to be prescriptive for the method in which data are transformed, but to provide a baseline for comparison and a means of cross referencing between data sets. Li applied the platform-specific analysis methods from R-ODAF to data generated from microarray, RNA-Sequencing, and Temp-O-Seq using identical samples with three biological replicates per treatment (Li et al. 2021). The output showed that RNA-Sequencing had the highest discovery rate of DEGs and microarrays the lowest. There were some DEGs in common but also a lot that were unique to each method within the limitations and specificities of each technology (Fig. 3). What we can be sure of in this analysis is that the differences arose from the capability of each of the analysis methods and not from the data transformation processes (see Fig. 1) as the R-ODAF was applied to each and those genes that were in common should be regarded as having the most robust relationship to the perturbation for initial toxicological analysis.

Finishing this section, von Bergen returned to AOPs to illustrate how they can be used to elegantly combine multiomics platforms, and demonstrated the utility of using several Omics methods to measure multiple KEs in sequence within an AOP.

#### Deriving PODs from Omics data

**Fig. 4:**
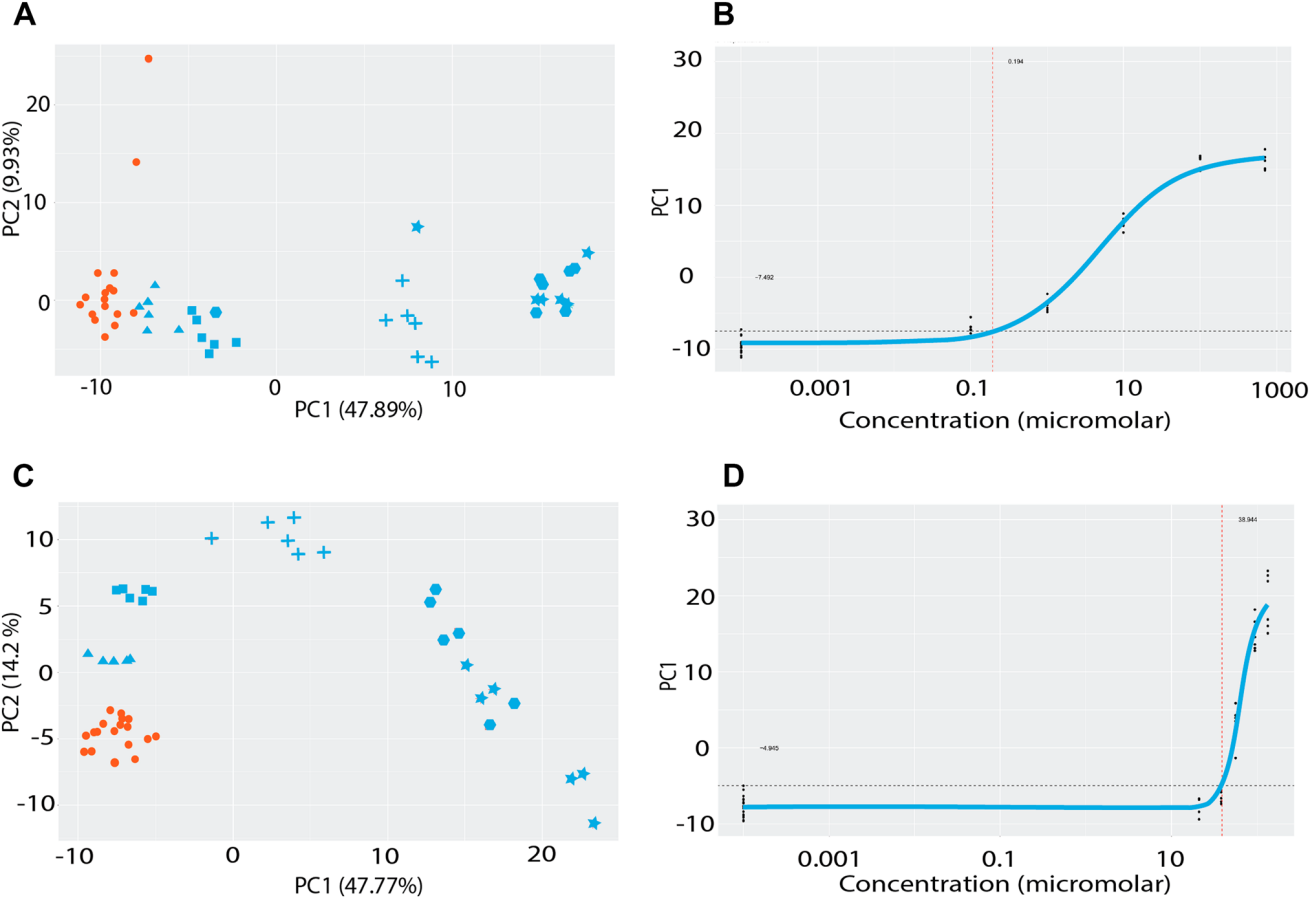
Examples of deriving PODs using principal component 1 for dose response modelling as described above for β- napthflavone (**A** and **B**) and aroclor (**C** and **D**). The PCA plots are shown in A and C and the derived POD plots in B and D. The control samples are the orange points and the treatment the blue points in increasing concentration from triangles, squares, crosses, hexagons and stars

The derivation of PODs from genomics data was covered by five speakers, Joshua Harrill (USEPA), Carole Yauk (University of Ottawa) and Scott Auerbach (National Institute of Environmental Health Sciences, USA) for transcriptomics, and Mark Viant (University of Birmingham UK) and Ben Ravenzwaay (Environmental Sciences Consulting, Germany) for metabolomics. Using extensive in vitro or in vivo case studies all presenters showed that Omics can be used to derive human health protective PODs. Harrill demonstrated how concentration-responsive transcriptomic effects measured in a small battery of in vitro models such as MCF7 breast carcinoma cells, U-2 OS osteosarcoma cells and differentiated HepaRG liver cells could be used to obtain tPODs which were comparable to or more sensitive than in vivo toxicity values for a majority of the several hundred chemicals evaluated. Concentration–response modelling of gene expression signatures could also be used to identify putative molecular targets and Molecular Initiating Events for certain chemicals (Harrill et al. 2021a). Auerbach had a similar approach using transcriptomic data but using a pathway-based approach. This work used a BMDExpress pathway package that is available from GitHub. The approach has been applied within a real-world setting to examine contamination of the Elk River by several substituted phenols. Transcriptomic data analysis suggested the potential for a high false discovery rate. One approach taken to address this was an in-silico analysis (resampling) of null data sets empirically estimating the false discovery rate. Yauk presented an analysis of 11 methods of selecting gene sets to derive transcriptomic PODs (Farmahin et al. 2017) for 6 chemicals using BMDExpress. The PODs for these diverse methods were generally within 3×, and all within 10× above or below apical POD’s (Farmahin et al. 2017). Further analysis demonstrated similar transcriptomic PODs by RNA-Sequencing, microarray and qPCR (Webster et al. 2015). Yauk also described a case study demonstrating how Omics can be used in a tiered-testing approach, where Tier 1 would encompass cell based high-throughput screening, Tier 2 Omics from short-term animal studies and conventional animal studies in Tier 3 (Gannon et al. 2019). A high concordance in predicted hazards from Tiers 1 and 2 were found in this study, highly aligned with Tier 3 outcomes, and with transcriptomic PODs correlated with the PODs derived from apical endpoint testing in Tier 3. ECETOC expanded on the simple tiered approach presented by Yauk with the inclusion of an in silico Tier 1 and the use of refined in vivo studies in the top Tier, which in this case was Tier 4, thus addressing reduction and refinement in addition to replacement. Viant demonstrated the use of metabolomics identified plasma biomarkers for the assessment of the POD for triphenylphosphate in a 5-day rodent assay. He was further able to demonstrate an additional use of metabolomics data in the identification of novel metabolites of triphenylphosphate. Van Ravenzwaay demonstrated a similar analysis using metabolomics signatures from in vitro experiments over an extended concentration range. Using β-naphthoflavone (Fig. 4A and B), a liver metabolizing enzyme inducer and Arochlor 1254 (Fig. 4C and D), an aryl-hydrocarbon receptor agonist examples, van Ravenzwaay was able to demonstrate that by using dose response modelling with the principal component 1 from the respective PCAs worked well for achieving a POD that could be used in regulatory assessment. The POD was derived using a twofold standard deviation from the control data. Interestingly, for Arochlor 1254 a dose response could also be seen in PC2, however, only at low concentrations. Theoretically, this could be the result of an initial (biochemical) response related to the Molecular Initiating Event and could serve as a potential discriminator between adaptive and adverse outcomes. Although care must be taken to reduce the false discovery rate, all of the studies in this section of the workshop demonstrated the strong potential for the use of Omics POD in chemical risk assessment, suggesting their suitability in a tiered approach.

#### Case study in using transcriptomics PODs in pharmaceutical development

A case study of the use of transcriptomic PODS in pharmaceutical development was presented by Frank Sistare. Sistare described 10 liver gene signatures that were indicative of various intracellular nuclear receptor activation and carcinogenic processes that were used for the stratification of internal drug candidates. Some of these have been recently published (Copple et al. 2021). These gene signatures are used for the refinement of conventional initial rat tolerability studies, which is an important contribution to reduction and refinement. Analysis of the aryl hydrocarbon receptor (AHR) gene signature in liver with 713 drug candidates indicated a statistical threshold (POD) of sevenfold for Cyp1A1 and 1.8-fold for Cyp1A2. Higher thresholds were derived for substances that were indicative of lower and higher tumorigenic concern (Qin et al. 2019). An important hypothesis was advanced that it is both the sustained activation of AHR and amplitude of the regulated genes that are important for carcinogenesis and that the transcriptional signatures and defined thresholds are useful for identifying this. The important finding for this section of the workshop though is that transcriptional signatures and threshold amplitudes could be used in short term assays using tissue collected from a routine study that identified substances and their associated dose levels of concern (Rooney et al. 2018).

### Output from the breakout groups

In framing the discussion for Day 2, the participants were reminded of the aims of the application of NAMs in chemical regulation, which are to overall protect public health by: (i) enhancing the pace of testing; (ii) ensuring data adequacy; (iii) increasing decision relevance; (iv) addressing the need to replace, reduce and refine, with an emphasis here not to forget the reduce and refine that is often overlooked in the rush to replacement; and (v) focusing on high tier human and environmental endpoint assessments. There were three breakout groups and each group considered all questions.

Question 1: “What is a relevant point of departure for different omics approaches and how is this accurately determined?”

To address this question, delegates considered that the first criterion for selecting a POD from Omics was that it should be relevant for the regulatory endpoint.

Discerning noise from response is an important consideration when selecting the measure used in the Omics analysis for the derivation of the POD. A persistent issue with Omics data from poorly designed studies is noise and associated false discoveries, as highlighted by several speakers. This issue is magnified if the data has a low number of replicates, which is often the case, or is being used at the lower end of the magnitude scale for fold change where there is more variance in the measurement of the changes in expression whether for transcripts, proteins or metabolites. Omics data, because of its high density have the potential for re-use in and in this context could for example be used in further studies where historical controls are required to establish normal variance and to suppress noise in data sets. Related to this point there was a consensus that positive controls for the adverse endpoint could be of benefit to the POD derivation and could be taken from historical data.

It was also noted that it is unwise to identify a POD by extrapolation too far below the lowest dose level tested (Gaylor 2012). In this respect, the discussants emphasized that dose levels need to span the range from no response to a dose having a large effect size (Kavlock et al. 1996; Kuljus et al. 2006; Program 2018; Sewell et al. 2022; Slob 2014).

For molecular POD determination, there is no current consensus on best practices. It was acknowledged that differences in sensitivities between Omics technologies assessing transcripts, proteins or metabolites and potentially in the shape of the dose responses according to the considered pathway or biological process can be observed. Nonetheless, non-linear regressions and mapping of gene-level data to gene sets using software such as BMDExpress is one potential method. If using outcome data, such as pathways, there may be a need to consider the biological relevance of the pathway to the apical outcome.

Methods that do not use pathways, for example using individual genes or BMD distributions, have also been suggested, as well as methods that do not use the BMD approach. The participants noted that it is important to examine the relative merits of each of these methods to identify a consensus process.

Question 2: “How do we determine biological significance/adversity of an omics response or ‘molecular mechanistic response’ (against a background of normal biological variation)?”.

Related to the question of what is a relevant POD in high dimensional omics data sets, the second question asked about biological significance of the molecular change underpinning the derived POD.

First, as classically used in toxicology, the treatment related nature of the molecular changes would need to be assessed to discriminate between the biological variability of the endpoints measured and the effects induced by the compound treatment. To expedite discussion in this workshop it was anticipated that monotonic dose responses of the treatment related molecular effects occurred. Moreover, the mechanistic linkage between the POD being considered and the adverse endpoint should be established. Consideration of the likely MoA could be used here in assessing relevance. This is particularly important when the adverse outcome may not be known for a particular substance; if identifying adversity in a molecular dataset is needed to derive a POD, there needs to be a plausible mechanistic linkage between the Omics POD and adverse outcome. It may also be necessary to consider whether the POD reflects homeostatic capacity that would resolve or a tipping point that leads to adversity. As Omics data offer the advantage of many molecular endpoints, it may be more expedient to consider more than one in determining adversity. This is consistent with the recommendations above to consider complex signatures, pathways or mathematical multivariate approaches. Finally, this breakout session considered that POD from traditional toxicity data could be useful to benchmark in vitro POD, though care will be necessary here to ensure that the Omics POD is causative.

Second, an alternative view is that identifying an adverse response from molecular data may not be needed for the regulatory goal of protecting human health. Determining adversity from molecular data requires prior qualitative (what) and quantitative (threshold value) knowledge about mechanisms and AOPs. Obtaining such knowledge is a long-term endeavour requiring a high amount of biological understanding. It may be that human health protection is afforded by defining the POD as the point at which “concerted” molecular change occurs (Johnson et al. 2022). Phenotypic changes to a biological system at the level of cells, organs, or organisms are the result of underlying system-level concerted molecular change. Such concerted molecular change is central to the individual gene methods outlined in the first question above.

Question 3: “What kind of experiment/data set would be necessary to identify non-adverse biological variation of controls?”

This question also arose within the preceding questions and so provoked less discussion. To identify effects that are deemed non-adverse, there is a need to understand the normal variance in the Omics and biological systems. This could be noise if technical but also normal biological variability. In either case, these changes might be statistically significant but not associated with an adverse outcome. For this reason, there is a need to understand the biological variability related to the system and its environment. These issues were also discussed in the previous questions; essentially, how stable is an Omics POD over time relative to its predicted adverse outcome and what ability does the organism have to overcome or adapt to this change without adverse consequence. Some historical in vivo data obtained in laboratory animals could be used to address this question.

Question 4: “Would mapping basic cellular responses in terms of their Omics-profile (or ‘molecular mechanistic data’) be helpful to better determine a true response from normal biological variation?”.

The connectivity mapping approach was raised as a way forward of dealing with this question (Lamb 2007; Lamb et al. 2006; Zhang and Gant 2009). This method may have advantages for several reasons. It determines relationships by reference to historical data and can use a statistical approach to determine the noise level in the data (Shah et al. 2022; Zhang and Gant 2008). In a similar manner to the other questions in the breakout session, it was recommended that prototype chemicals be characterized in MoA framework-based studies (dose /time concordance, reversibility) on target and non-target organs to gain a deeper understanding of the molecular basis of diverse adverse outcomes. In terms of using a pathway approach, the major issue with this method was identified in that it usually relies on a mathematical approach to consider whether a pathway is activated and does not consider rate limiting steps that need to be incorporated to make the most use of this approach.

An alternative view was that mapping (i.e., mechanistic knowledge) of Oomics data may not be necessary to identify a true biological response from background noise. In this case, understanding the molecular population at baseline and the range of normal variation within the population at baseline may be all that is needed. Such information could be gathered by establishing a community-wide database of molecular data from historical control samples and/or generating baseline molecular data from a variety of control sample types. Technical analysis of these control datasets might be used to establish the range of values present in a baseline control population. This information could then be used to establish methods to identify when a change at the molecular level is likely to be due to chemical exposure.

## Discussion and conclusions

It has been more than 20 years since the development of Omics methodologies (Burge 2001). While these methods have found application internally in companies for triaging and in some limited respects in regulatory submissions to support mechanistic associations, there is still progress to be made in reaching a point of routine regulatory application to address the 3Rs of refinement, reduction, and replacement of animals used in research. This is disappointing as these methods have so much to offer to the 3Rs and have the potential to generate more protective regulatory assessments for human and environmental health. Lack of uptake has occurred despite substantial investment in projects to develop these methods in the US, Canada and the EU. An underpinning issue is data confidence, though some Omics-based biomarkers have been approved such as MammaPrint^®^ and GARDskin^®^ (Glas et al. 2006; Knauer et al. 2010; Senzagen 2022). This lack of Omics data use in regulatory practice was recognized by ECETOC in 2015 and led to the initiatives that developed the OORF and ODAF (Fig. 1) (Harrill et al. 2021b; Verheijen et al. 2022). Many of these methods have been taken to the international community through the OECD, and it is hoped in time will be incorporated into Guidance Documents and finally Test Guidelines that will assure the Mutual Acceptance of Data.

In this workshop, the progress over twenty years in genomics was highlighted with many of the participants having been in the field for all of the last two decades. The breakout sessions explored several key questions, and the results demonstrated significant progress made. However, there remain pressing experimental and bioinformatic issues that have hindered regulatory adoption; some of these issues are addressed through the development of the OORF and ODAF. These efforts by the OECD are advancing the use of Omics in regulatory testing either alone or as part of integrated approaches to testing and assessment (IATAs). These initiatives aim to increase the transparency, robustness and reproducibility of omics data in general.

Of note though for future work is that these activities do not address the toxicological interpretation component. The importance of filling this gap was reflected in this workshop, with discussion about: (i) the potential need to understand the relationship of the whole Omics data set and individual components thereof to the adverse outcome; (ii) the influence of biological variability (also termed ‘noise’); and (iii) the discernment of changes that are causative and not simply a result of altered pathophysiology. This workshop discussion identified necessary work to advance the interpretation of Omics data in particular as it relates to POD determination. The hope is that these recommendations will be subject to consideration within organizations including the OECD. This international consideration is needed to arrive at consensus on the best approaches to application of Omics data in regulatory testing to build confidence in its use and capitalize on tools to address the 3Rs.

In conclusion, in 20 years the technical generation and bioinformatic processing of Omics data have progressed substantially, and promising applications to inform decision making have been demonstrated through a plethora of NAM projects and case studies. This workshop highlighted the significant advances made and articulates the issues that remain to be resolved to progress further. These key issues relate primarily to the biological and toxicological understanding/interpretation of Omics data sets, rather than the technical steps involved in generating or processing the data. Addressing these issues would progress the inclusion of these protocols at least in the initial tiers of regulatory testing. There is now a need to refine and translate that knowledge into internationally accepted protocols that can be used in chemicals regulation. Achieving this has been and will continue to be the goal of ECETOC and its partners, with an aim to first reduce and then replace animals in chemical testing.

## Data Availability

Requests for data used in the preparation of this article can be sent to the corresponding author.

## Abbreviations

AOP: Adverse outcome pathway
cDNA: Copy deoxyribonucleic acid
DAPRM: Data acquisition and processing report modules
DEGs: Differentially expressed genes
EAGMST: Extended Advisory Group on Molecular Screening and Toxicogenomics
ECETOC: European Centre for Ecotoxicology and Toxicology of Chemicals
IATAs: Integrated approaches to testing and assessment
KE: Key biological events
KER: Key Event Relationship
MIE: Molecular initiating event
MoA: Mode of action
MRF: Metabolomics reporting framework
NAMs: New approach methodologies
NMR: Nuclear magnetic resonance
ODAF: Omics data analysis framework
OORF: OECD Omics reporting framework
PoD: Point of departure
qPCR: Quantitative polymerase chain reaction
RNA: Ribonucleic acid
TempO-Seq: Templated oligo-sequencing
tPOD: Transcriptomic Point of Departure
TRF: Transcriptomics reporting framework
US EPA: US Environmental Protection Agency
